# Supplementary material for: Winter temperature predicts prolonged diapause in pine processionary moth species across their geographic range
Source: PeerJ. 2019 Feb 28;7:e6530. doi: 10.7717/peerj.6530 (PMC6397759; doi:10.7717/peerj.6530)
Supplement: Data S1 — Each data point contain information related to: Year, Country, Site, Latitude, Longitude, Elevation, Host, Clade, Sub-Clade, T mean cold period °C, No. of individuals, Condition, Mortality %, Prolonged diapause %, Reference. [file peerj-07-6530-s001.docx]

Supplemental file S1

Winter temperature predicts prolonged diapause in pine processionary moth across its geographic range

Md H.R. Salman et al.

Submission to PeerJ

List of the sites

| **Country** | **Site** | **Latitude** | **Longitude** | **Elevation (m)** | **Years of sampling** | **References** |
| --- | --- | --- | --- | --- | --- | --- |
| Algeria | Bou-Saâda | 35.149825 | 4.075044 | 883 | 1983 | Bertella (1987) |
| Algeria | Ain-Yagout | 35.772556 | 6.410947 | 895 | 1983 | Bertella (1987) |
| Algeria | Bouilef | 35.58086 | 6.20339 | 1100 | 1983 | Bertella (1987) |
| Algeria | Ich-Ali | 35.519619 | 6.128136 | 1146 | 1983 | Bertella (1987) |
| Algeria | Moudjebara | 34.62361 | 3.32389 | 1210 | 1994-1995 | Zamoum (1998) |
| Algeria | Senalba Chergui | 34.64944 | 3.19528 | 1253 | 1994-1995 | Zamoum (1998) |
| France | Berder | 47.575 | -2.89 | 10 | 1972-1977 | Abgrall (2001) |
| France | Les Mathes-La Palmyre | 45.69861 | -1.19778 | 10 | 1971-1974 | Abgrall (2001) |
| France | Vielle St Girons | 43.95528 | -1.34389 | 20 | 1971-1977 | Abgrall (2001) |
| France | Landes de Gascognes | 44.73333 | -0.76667 | 59 | 2008 | Dulaurent et al. (2011) |
| France | Vienne en Val | 47.8057611 | 2.120861 | 102 | 2015 | Laparie (2016) |
| France | Marcillac-en-Vallon | 44.478181 | 2.482817 | 330-400 | 2014 | Laparie (2016) |
| France | St Germain de Calberte | 44.21889 | 3.80361 | 600 | 1972-1977 | Abgrall (2001) |
| Greece | Thessaloniki | 40.63556 | 22.98278 | 247 | 1984 | Markalas (1989) |
| Israel | Ramat Gan | 32.06583 | 34.83139 | 50 | 1970 | Halperin  (1990) |
| Israel | Eshtaol | 31.78278 | 34.9925 | 360 | 1964 | Halperin (1969) |
| Israel | Ramallah | 31.90333 | 35.21083 | 850 | 1969 | Halperin  (1990) |
| Italy | Calabria | 38.0694444 | 15.82722 | 1200 | 2010-2012 | Bonsignore (2016) |
| Italy | Colle Calbarina | 45.27306 | 11.73722 | 136 | 1993-1996 | Ghiraldo (1994) Ardesi (1996) |
| Italy | Venosta | 46.6275 | 10.80306 | 822-910 | 1999-2015 | Salman (2016) |
| Morocco | Oued Beth | 33.88028 | -5.91639 | 250 | 1988 | Graf (1988) |
| Morocco | Tiflet | 33.900889 | -6.303508 | 300 | 1988 | Graf (1988) |
| Morocco | Ain Rami | 35.1325 | -5.28222 | 500 | 1988 | Graf (1988) |
| Morocco | El Ghomra | 34.11722 | -4.96889 | 500 | 1988 | Graf (1988) |
| Morocco | Saka | 34.613783 | -3.417656 | 750 | 1988 | Graf (1988) |
| Morocco | J'Zerhoun | 34.03944 | -5.50361 | 800 | 1988 | Graf (1988) |
| Morocco | Tafoughalt | 34.80889 | -2.40306 | 800 | 1988 | Graf (1988) |
| Morocco | Bab Taza | 35.06111 | -5.21194 | 820 | 1988 | Graf (1988) |
| Morocco | Ain B.Mathar | 34.010842 | -2.030275 | 900 | 1988 | Graf (1988) |
| Morocco | Jerrada | 34.29167 | -2.155 | 1000 | 1988 | Graf (1988) |
| Morocco | Tahanaout | 31.324689 | -7.947678 | 1000 | 1988 | Graf (1988) |
| Morocco | Bouhachem | 35.24889 | -5.42389 | 1157 | 1988 | Graf (1988) |
| Morocco | Issaguen | 34.91361 | -4.57556 | 1548 | 1988 | Graf (1988) |
| Morocco | Boutroubay | 33.45028 | -5.04222 | 1910 | 1988 | Graf (1988) |
| Spain | S. Calderona | 39.69722 | -0.54333 | 300 | 2000-2001 | Lopez (2014) |
| Spain | Los Serranos | 39.93111 | -1.06944 | 1238 | 2000-2001 | Lopez (2014) |
| Spain | La Cortijuela | 37.10806 | -3.4825 | 1600 | 2010 | Torres-Muros et al. (2017) |

Description of the studies used for the analysis

**Algeria**

**Bertella (1987): Batna region**

Bertella N. 1987. Bio-ecologie de *Thaumetopoea pityocampa* Schiff. (Lepidoptera, Thaumetopeidae) dans quelques régions d’Algérie. Thèse magister Sci. Agr.., Institut National Agronomique El Harrach, Algérie, 110 pp.

- **Temperature data**

Retrieved from the document (weather station Batna).

- **Sampling**

One cohort (1983) and two types of experiments.

1. Larvae followed in the field

During the procession period, in April 1983, larvae were followed in the field (2 sites: plantation of Bouilef and plantation of Chaâba) and the places where they entered the soil were recorded, the number of larvae in the colony was counted before they started to enter the soil. The pupae were taken to the lab 3 months after the burying. Pupae were conserved individually in tubes (14x1.6 cm).

The number of pupae found, the number of moths emerged in the current year, the number of pupae in prolonged diapause and the number of pupae parasitized and attacked by fungi, empty cocoons and other conditions were recorded. The rate of emergence was calculated from the number of pupae found 3 months after entering the soil.

This sample may include individuals that were already in the soil from previous cohorts.

1. Larvae brought to Batna and put in pots

During the procession period, in April 1983, larvae were collected in the field (at 5 sites: plantation along the road Djelfa-Bou-saâda, plantation of Ich Ali, Chaâba, Bouilef and Ain Yagout all in the Batna Region) and put in pots filled with soil in Batna.

Larvae were put in pots by colony and by plantation. Pots were black, cylindrical and in plastic (16x52 cm). Each pot was composed of 2/3 of soil and 1/3 of sand and was numbered. All pots were taken in a hangar ventilated and covered at Batna. A cloth was put on the pots in order to catch the adults after emergence. Pupae still in soil in pots were taken after total emergence of the current year adults, and the pupae in prolonged diapause were kept in lab in tubes.

The number of larvae put in pots was counted as well as the number of dead larvae, the number of dead pupae, the number of parasitised pupae, the number of moths emerged in the current year and the number of pupae in prolonged diapause.

This sample include individuals of the 1983 cohort only.

1. Larvae followed in the field

| Site | Larvae | Pupae | Emergence current year | % Emergence current year | Prolonged diapause | % Prolonged diapause | Pupae parasitised | Pupae affected by fungi | Empty cocoons | Other forms | % Mortality^a^ |
| --- | --- | --- | --- | --- | --- | --- | --- | --- | --- | --- | --- |
| Bouilef | 453 | 263 | 151 | 56.3 | 21 | 8.0 | 51 | 6 | 16 | 18 | 34.7 |
| Chaâba | 482 | 294 | 181 | 61.6 | 31 | 10.5 | 44 | 17 | 12 | 9 | 27.9 |

a: Mortality calculate by us, in the discussion of the thesis it is reported 14.8% in Bouilef and 14.4% in Chaâba with reference to pathogens, although it is not clear from the data.

1. Larvae brought to Batna and put in pots

|  |  |  | Number of pupae | | | | |  | % Pupae | | |
| --- | --- | --- | --- | --- | --- | --- | --- | --- | --- | --- | --- |
| Site | Number larvae | Number dead larvae | Dead | Parasitised | Attacked by fungi | Emergence n | Emergence n+1 | % Dead larvae | Dead/parasitised/fungi | Emergence n | Emergence n+1 |
| Ich-Ali | 196 | 25 | 26 | 20 | 12 | 84 | 29 | 12.8 | 29.6 | 42.9 | 14.7 |
| Ain-Yagout | 176 | 12 | 51 | 18 | 11 | 78 | 6 | 6.8 | 45.5 | 44.3 | 3.4 |
| Bou-Saâda | 229 | 23 | 33 | 15 | 16 | 123 | 19 | 10 | 28 | 53.7 | 8.3 |
| Bouilef | 244 | 40 | 49 | 23 | 12 | 103 | 17 | 16.4 | 34.3 | 42.2 | 7 |

- **Analysis**

1. Larvae followed in the field

There were a low number of pupae found in the soil compared with the number of larvae buried. It was explained by the fact it was natural buried so the larvae were “free” to change the location of buried, the parasitism was also a reason and finally the mistake during the counting.

The rate of emergence the current year was 56.3% in Bouilef and 61.6% in Chaâba. The % of diapause was 8 in Bouilef and 10.5 in Chaâba. And the mortality mostly due to parasitism was 34.7% in Bouilef and 27.9% in Chaâba.

1. Larvae brought to Batna and put in pots

The rate of larval mortality was around 11.8% (mean for all sites). The rate of emergence the current year was 46% for all the pupae, with a maximum of 53.7% in Bou-saâda. The rate of parasitism was 13.6% in Bou-saâda and up to 16.5% in Ain-Yagout. The rate of prolonged diapause was from 3.4 (in Ain-Yagout) to 14.7% (in Ich-Ali).

The plantation in Ich-Ali was faced to the North and it was the only difference with the plantation of Bouilef (same elevation 1000 to 1040 m); in Bouilef the rate of prolonged diapause was just 7%. Ain-Yagout and Bou-saâda are located on the plain. The rate of mortality for all the sampling was 19% and can be explained by the hot temperature during April emphasized by the plastic pots. The rates of parasitism and attack by fungi were very low; indeed the pupae were isolated from their natural site.

**Zamoum (1998): Djelfa region**

Zamoum M. 1998. Données sur la bioécologie, les facteurs de mortalité et la dynamique des populations de *Thaumetopoea pityocampa* Denis et Schiffermüller (Lep., Thaumetopoeidae) dans les pineraies subsahariennes de la région de Djelfa (Algérie). Thèse de Doctorat, Univ. des Sciences de Rennes I, France, 247 pp.

- **Temperature data**

Retrieved from the document (weather station Djelfa).

- **Sampling**

The pupae of both samplings were check daily to evaluate the emergence period of *T. pityocampa* adults and mortality factors, including parasitoids.

For the purpose of comparison with other sites, data from M1, MN, M3 and M4 were pooled for Moudjebara and S1, S2 and S3 for Senalba Chergui.

1. Data from Moudjebara (M) and Senalba Chergui (S) (% emergence and % diapause calculated from the number of healthy pupae. The % of mortality calculated from the number of larvae at the beginning.)

a: cohort 1994 b: cohort 1995

|  |  |  |  |  |  | % e |  |  |  |  |  |  |  | % d |  | %m |  |
| --- | --- | --- | --- | --- | --- | --- | --- | --- | --- | --- | --- | --- | --- | --- | --- | --- | --- |
| Plots | Elevation | N. larvae ^a^ | N. larvae ^b^ | N.  adults ^a^ | N. adults ^b^ | n ^a^ | n ^b^ | n+1 ^a^ | n+1 ^b^ | n+2 ^a^ | n+2^b^ | n+3 ^a^ | n+3 ^b^ | a | b | a | b |
| M1 | 1340 | 661 | 835 | 345 | 365 | 95.1 | 84.9 | 4.3 | 10.4 | 0.3 | 4.7 | 0.3 | 0 | 4.9 | 15.1 | 47.8 | 56.3 |
| MN | 1180 | 762 | 1054 | 493 | 667 | 99.2 | 90 | 0.8 | 6.1 | 0 | 3.9 | 0 | 0 | 0.8 | 10 | 35.3 | 36.7 |
| M3 | 1200 | 547 | 1616 | 129 | 742 | 87.6 | 84.8 | 10.1 | 11.1 | 2.3 | 4.2 | 0 | 0 | 12.4 | 15.2 | 76.4 | 54.1 |
| M4 | 1120 | 594 | 1492 | 328 | 254 | 96.6 | 94.1 | 3.4 | 3.1 | 0 | 2.8 | 0 | 0 | 3.4 | 5.9 | 44.8 | 83 |
| S1 | 1280 | 460 | 522 | 181 | 350 | 97.2 | 84.3 | 2.8 | 11.4 | 0 | 4.3 | 0 | 0 | 2.8 | 15.7 | 60.7 | 33 |
| S2 | 1240 | 516 | 598 | 300 | 436 | 99.7 | 75.5 | 0.3 | 17.9 | 0 | 6.7 | 0 | 0 | 0.3 | 24.5 | 41.9 | 27.1 |
| S3 | 1240 | 244 | 663 | 104 | 214 | 100 | 61.2 | 0 | 22 | 0 | 16.8 | 0 | 0 | 0 | 38.8 | 57.4 | 67.7 |

1. Data from Moudjebara (% emergence and % diapause calculated from the number of observed adults. The % of mortality calculated from the number of larvae at the beginning.)

a: cohort 1995 b: cohort 1996

| Plot | Elevation | Treatment | Number larvae ^a^ | Number larvae ^b^ | Number pupae ^a^ | Number pupae ^b^ | Number adults ^a^ | Number adults ^b^ |
| --- | --- | --- | --- | --- | --- | --- | --- | --- |
| M1 | 1340 | Sun | 400 | 74 | 380 | 72 | 243 | 56 |
|  |  | Partial Shade | 266 | 200 | 245 | 147 | 119 | 90 |
|  |  | Shade | 459 | 74 | 423 | 73 | 87 | 20 |

|  | % Emergence | | | | | | | | % Prolonged diapause | | % Mortality | |
| --- | --- | --- | --- | --- | --- | --- | --- | --- | --- | --- | --- | --- |
| Treatment | n ^a^ | n ^b^ | n+1 ^a^ | n+1 ^b^ | n+2 ^a^ | n+2 ^b^ | n+3 ^a^ | n+3 ^b^ | a | b | a | b |
| Sun | 34.7 | 87.2 | 62.3 | 12.8 | 2.1 | 0 | 0.8 | 0 | 65.3 | 12.8 | 39.3 | 24.3 |
| Partial Shade | 27.9 | 62.5 | 69.4 | 37.5 | 2.7 | 0 | 0 | 0 | 72.1 | 37.5 | 55.3 | 41.5 |
| Shade | 23.5 | 47.1 | 75.3 | 47.1 | 1.2 | 5.9 | 0 | 0 | 76.5 | 52.9 | 81 | 73 |

- **Analysis**

1. Larvae from processions at the base of trees

The rate of survival and diapause were analysed, after a X^2^ the results showed a varying survival rate between years (except in MN and S2). For both years, the rate of survival was between 17% (M4 in 1995) and 72.9% (S2 in 1995). The emergence was mostly annual, with a higher rate in 1994 (87.6%-100%) than in 1995 (61.2%-94.1%).

The rate of prolonged diapause was higher in 1995 (5.9% in M4 and 38.8% in S3) than in 1994 (0% in S3 and 12.4% in M3). These rates varied from year to year and from plot to plot.

The larvae predation, very low (0.3% in MN to 0.7% in M4 both in 1994), was caused by *Scolopendra sp*.

The rate of parasitism was between 4% (M4 in 1995) and 42.2% (S3 in 1995). These rates were higher in M1, S1, S2 and S3 (plot close to natural forest).

The rate of unknown mortality was from 6.5% (S2 in 1995) to 72% (M4 in 1995). Mycosis accounted up to 5.4% (M1 in 1994) and overall mortality caused by diseases was from 0.4% (S3 in 1994) to 4% (M3 in 1994 and 1995).

1. Pupae from the ground

Same analyses as for the first sampling. The mortality was between 27.8% and 82% in 1994 and between 27% and 83% in 1995. Mortality varied from year to year (X^2^=41.09, p<0.001). The rate of survival, both years is higher in sunny sites (60%) and lower in shaded sites (max 19%).

A comparison between the results from the two types of sampling was done. The rate of prolonged diapause was higher for the larvae pupating in the natural soil (up to 86%) than for the larvae put in tubes (up to 39%). In addition, in the sampling 2 the pupae stayed into diapause up to 3 years while only 1 year was observed for sampling 1.

The maximum duration of diapause recorded was 6 years in Djelfa and 3 years for other sites in Algeria, although a precise estimation about the number of individuals emerging in the different years is not available.

**France**

Abgrall, J.F., 2001. Le reseau surveillance processionnaire du pin en France 1969-1989. Conception – Historique – Résultats. CEMAGREF, Direction de l’Espace rural et forestier, Nogent sur Vernisson.

1. ***Ile aux Moines*** (Abgrall 2001)

- **Temperature data**

Retrieved from <http://meteo-climat-bzh.dyndns.org> (weather station Vannes 47°36'00"N, 02°42'36"W, 3m).

- **Sampling**

Caterpillar were sampled during the procession and isolated within a rhodoïd cylinder. These cylinders were driven to the ground for 2 to 3 cm, in open areas. Caterpillars were then put inside. At the right period, these cylinders were removed and the soil was taken off for 15 to 30 cm in order to find cocoons. After that, the cocoons were taken to the laboratory.

| Site | Elevation (m) | Number pupae | Year of sampling | % Emergence (n) | % Diapause | % Mortality | % Parasited pupae |
| --- | --- | --- | --- | --- | --- | --- | --- |
| Ile aux moines | 11 | / | 1971 | 90.54 | 7.2 | 0.9 | 1.36 |

- **Analysis**

The point was to make a survey of the region to decide if it was necessary to treat the area. For that they recorded the level of infestation, the date of emergence and the rate of emergence the first year, diapause and mortality. There was a very high rate of emergence the first year with 90.54% and a very low rate of mortality and parasitism with 0.9% and 1.36%, respectively. The rate of diapause was 7.2%. There was no survey of the diapausing pupae over years.

1. ***Berder*** (Abgrall 2001)

- **Temperature data**

Retrieved from <http://meteo-climat-bzh.dyndns.org> (weather station Vannes 47°36'00"N, 02°42'36"W, 3m).

- **Sampling**

Larvae of pine processionary moth were collected during the procession and then isolated with a rhodoïd cylinder. These cylinders were driven to the ground for 2 to 3 cm, in open areas. At the right period, these cylinders were removed and the soil was taken off for 15 to 30 cm in order to find cocoons. After that, the cocoons were taken to the laboratory.

| Site | Elevation (m) | Number pupae | Year of sampling | % Emergence (n) | % Diapause | % Mortality | % Parasited pupae |
| --- | --- | --- | --- | --- | --- | --- | --- |
| Berder | 10 | 169 | 1972 | 85.6 | 5.9 | 8.5 | 0 |
|  |  | 45 | 1973 | 73.3 | 2.22 | 0.03 | 24.45 |
|  |  | 164 | 1974 | 51.21 | 0 | 11.58 | 37.19 |
|  |  | 47 | 1977 | 93.61 | 0 | 4.25 | 2.12 |

- **Analysis**

The rate of emergence was generally high with a rate from 73.3% (in 1973) to 93.61% (in 1977). But there was an exception in 1974, where the rate was just 51.21%. It was explain by a higher mortality and parasitism this year with 11.58% of mortality and 37.19% of parasitism. The rate of diapause was from 0 (in 1974 and 1977) to 5.9% (in 1972).

In September 1971, there was a microbiologic treatment (solution of *Bacillus thuringiensis*). Considering the level of infestation in 1972, the treatment was not effective and another protocol was recommended. In 1973, the level of infestation was lower and the population was considering stable. In 1974, the infestation was growing again and consequently a microbiologic treatment was advised on the third larval instar. In 1977, the rate of diapause was null and the mortality and the parasitism were very low that indicated an increasing population density, so a treatment was recommended.

1. ***Les Mathes-La Palmyre*** (Abgrall 2001)

- **Temperature data**

Retrieved from <http://meteo-climat-bzh.dyndns.org> (weather station Vannes 47°36'00"N, 02°42'36"W, 3m).

- **Sampling**

In 1971 and 1972, the sampling was done at Bonne Anse (The Mathes) and from 1973 to 1975, the sampling was done at La Palmyre. Larvae of pine processionary moth were collected during the procession and then isolated with a rhodoïd cylinder. These cylinders were driven to the ground for 2 to 3 cm, in open areas. At the right period, these cylinders were removed and the soil was taken off for 15 to 30 cm in order to find cocoons. After that, the cocoons were taken to the laboratory.

| Site | Elevation (m) | Number pupae | Year of sampling | % Emergence (n) | % Diapause | % Mortality | % Parasited pupae |
| --- | --- | --- | --- | --- | --- | --- | --- |
| Les Mathes-La Palmyre | 10 | 42 | 1971 | 66.7 | 28.9 | 2.2 | 2.2 |
|  |  | 52 | 1972 | 48.0 | 38.0 | 1.9 | 11.5 |
|  |  | 199 | 1974 | 75.4 | 13.1 | 0.5 | 11.0 |

- **Analysis**

The emergence varied from 48% (in 1972) to 75.37% (in 1974). The mortality was low all the years (0.5% in 1974 to 2.22% in 1971). The parasitism varied from 2.22% in 1971 to 11.5% in 1972. The rate of diapause was from 13.06% in 1974 and up to 38% in 1972.

In 1971, the rate of diapause was explained by the climatic conditions, a treatment was advised for September 1971. In 1972, the level of infestation was lower thanks to the treatment, no treatment was planned. In 1974, the infestation was in progression but the lower rate of diapause showed that the population became quantitatively stable.

1. ***Vielle Saint Girons*** (Abgrall 2001)

- **Temperature data**

Retrieved from <http://meteo-climat-bzh.dyndns.org> (weather station Mont de Marsan (43°55'12"N, 00°30'00"W, 60m).

- **Sampling**

Larvae of pine processionary moth were collected during the procession and then isolated with a rhodoïd cylinder. These cylinders were driven to the ground for 2 to 3 cm, in open areas. At the right period, these cylinders were removed and the soil was taken off for 15 to 30 cm in order to find cocoons. After that, the cocoons were taken to the laboratory.

| Site | Elevation (m) | Number pupae | Year of sampling | % Emergence (n) | % Diapause | % Mortality | % Parasited pupae |
| --- | --- | --- | --- | --- | --- | --- | --- |
| Vielle Saint Girons | 20 | 91 | 1971 | 83.52 | 8.8 | 2.19 | 5.49 |
|  |  | 88 | 1972 | 62 | 3.4 | 0 | 35 |
|  |  | 235 | 1974 | 80 | 2.9 | 0 | 17 |
|  |  | 309 | 1975 | 29.77 | 12 | 20 | 38 |
|  |  | 240 | 1976 | 85.83 | 3.3 | 0.83 | 10 |
|  |  | 251 | 1977 | 76.49 | 14.34 | 2.39 | 6.71 |

- **Analysis**

The rate of emergence varied from 29.77% in 1975 to 85.83% in 1976. The mortality is low except in 1975 (20%). The parasitism was from 5.49% in 1971 to 38% in 1975. The diapause varied from 2.9 in 1974 to 14.34% in 1977. There was a treatment each year. No explanation regarding the low rate of emergence in 1975.

1. ***Saint Germain de Calberte*** (Abgrall 2001)

- **Temperature data**

Retrieved from <http://meteo-climat-bzh.dyndns.org> (weather station Nimes (43°51'30"N, 04°24'24"E, 59 m).

- **Sampling**

Larvae of pine processionary moth were collected during the procession and then isolated with a rhodoïd cylinder. These cylinders were driven to the ground for 2 to 3 cm, in open areas. At the right period, these cylinders were removed and the soil was taken off for 15 to 30 cm in order to find cocoons. After that, the cocoons were taken to the laboratory.

| Site | Elevation (m) | Number pupae | Year of sampling | % Emergence (n) | % Diapause | % Mortality | % Parasited pupae |
| --- | --- | --- | --- | --- | --- | --- | --- |
| Saint Germain de Calberte | 600-800 | 100 | 1972 | 78 | 17 | 2 | 3 |
|  |  | 642 | 1974 | 88.76 | 7.75 | 1.1 | 2.37 |
|  |  | 100 | 1975 | 89 | 0 | 10 | 1 |
|  |  | 231 | 1977 | 85.38 | 0 | 2.16 | 12.55 |

- **Analysis**

The rate of emergence of emergence diapause, mortality and parasite pupae were recorded for the first year. The rate of emergence varied from 78% (in 1972) to 89% (in 1975). The rate of diapause varied from 0% (in 1975 and 1977) to 17% in 1972. The rate of mortality is very low, from 1.1% (in 1974) to 10% (in 1975). For the parasitism of the pupae, the rate was from 1% (in 1975) to 12.55% (in 1977).

In 1972, the 17% of pupae in diapause were still in survey in order to see the emergence, diapause and mortality at n+1, i.e. in 1973. On the 17% of diapausing pupae, 70.6% emerged in 1973, 11.76% were still in diapause, 0% was dead and 17.65% were parasitised. Treatment was applied in 1972.

1. ***Landes de Gascogne (Dulaurent et al. 2011)***

Dulaurent, A.M., Porté, A.J., van Halder, I., Vétillard, F., Menassieu, P., Jactel, H., 2011. A case of habitat complementation in forest pests: pine processionary moth pupae survive better in open areas. For. Ecol. Manage. 261, 1069-1076.

- **Temperature data**

Retrieved from <http://meteo-climat-bzh.dyndns.org> (weather station Bordeaux-Merignac 44.83°N, 0.69°W, 47 m).

- **Sampling**

Cohort 2008. In March 2008, 3062 pine processionary caterpillars in procession were collected on several forest tracks close to the experimental site. In each procession, 10% of the caterpillars were sampled and kept under laboratory conditions to evaluate the parasitism rate before the pupation stage. The rest of the caterpillars were split into 54 groups of 50 caterpillars. The groups were composed of caterpillars from different processions to have genetic heterogeneity. The groups of caterpillars were placed on the soil surface of the bucket and the buckets were covered with a thin wire netting. In mid-September, the buckets were collected and checked layer by layer to retrieved the 50 pupae. As the 50 pupae could not be found, the number of vanished cocoons was calculated (*V*) (50 – total number of retrieved cocoons). For the retrieved cocoons, 5 categories of pupae: (*E*) emerged, (*P*) parasitized, (*F*) infected by fungi, (*C*) failed to pupate/dead caterpillars, (*D*) diapausing pupae.

So for each bucket: *E* + *D* + *V* + *P* + *F* + *C* = 50. The mortality rate was calculated as the sum of the four categories of mortality: M% = (V + P + F + C)/50.

- **Analysis**

The point of this experiment was to test three hypothesis: (1) The survival of the PPM pupae is higher in open areas than under pine and broadleaved forest covers. (2) Survival rates of PPM pupae related to micro-climatic soil conditions that depend on both forest cover type and soil origin. (3) Open areas provide complementary habitats to PPM individuals, whereas broadleaved stands may act as ecological traps.

They tested 2 types of classification: the *a priori* classification (3 classes of land cover and soil (see above)) and the *a posteriori* classification (2 classes of land cover and soil: Cover type = Open/Forest; Soil type = Non Pine/Pine). Then they analysed the effect of land cover types and soil origins on the percentage of pupae and the mortality rate and they analysed the proportion of emerged and diapausing pupae with the microclimatic variables.

Using the *a priori* classification of habitat conditions, the rate of emergence varied from 37% to 62.7%. The rate of mortality varied from 36.7% to 57.3% (with V=17.5±1.0%, F=17.4±0.9%, C=6.3±0.7%, P=6.1±0.6%) and the rate of diapause varied from 0.7% to 9%.

The rate of emergence was higher significantly in open area whereas the rate of mortality and diapause were lower in open area compared to pine or broadleaved tree covers.

Mortality was higher in the soil of pine forest compared to the soil of broadleaved forest.

Using the *a posteriori* classification, the effect of the forest cover on the rate of emergence, diapause and mortality was significant, it was more significant than with the *a priori* classification.

1. **Unpublished data (Mathieu Laparie INRA)**

- **Temperature data**

Measured on site by dataloggers.

- **Sampling sites**

Three distinct populations were investigated and reported in the present document: Marcillac-en-Vallon 2013-2014), Vienne-en-Val 2014-2015. We sampled additional populations in different sites or years, but encountered experimental failures that prevented proper analysis of diapause and mortality rates.

- **Sampling**

*i. Écopièges*

We installed Écopièges traps on 20 attacked trees in Marcillac-en-Vallon at the end of March 2014, and on 36 attacked trees in Vienne-en-Val in early March 2015. The number of presumably inhabited tents in each tree was noted. Each trap was customized to our needs and consisted in a collar around the trunk preventing larvae to reach the ground during their pupation procession, connected to a geotextile bag of sand buried 30 cm into the ground. The bag was connected to the collar by two standard Écopiège plastic pipes. Within the collar, the upper end of both pipes were separated by a 5 cm high plastic wall to limit larvae crawling, and instead stimulate them into finding an exit next to the pipes, thereby accelerating their descent into the bag.

In Marcillac-en-Vallon, traps were left until late May 2014, *i.e.*, after the usual procession period, to make sure that a maximum of bags would contain pupae. They were immediately brought back to the laboratory in Orléans and sorted to count dead and living pupae. Every pupae still alive was individualized in an assay glass tube capped with cotton and kept in the dark at 20 ± 2 °C, except for bags containing >60 pupae still alive, in which pupae were subdivided into two groups: one for the laboratory in the above conditions, and one for outdoor monitoring. Pupae that belonged to the latter group were transferred back into their geotextile bag containing sand and buried 30 cm deep into the ground at INRA (Orléans) for later emergence monitoring.

Vienne-en-Val being situated close to the laboratory, the traps could be monitored regularly to check for signs of procession inside the collars (silk tracks, feces, or even direct observation of pupating individuals in the geotextile bags). Firsts signs of procession appeared as early as March 9^th^ in some traps (except one where larvae were observed crawling a couple hours after the trap was installed on March 1^st^), but signs of activity were observed in other traps until the end of March. Bags were left untouched until June to make sure that pupation could proceed without further manipulation and perturbation, since individuals are particularly fragile and vulnerable during pupation. The sand in each bag was eventually transferred into a pot capped with mesh netting, and left outside under shelter.

*ii. Feeding of late colonies in pots*

In Corsica, no regular field trips to install, check and then collect traps were possible. We therefore used an alternative method by chopping 22 tents off attacked trees at the end of the larval development (April 2015, during the procession period (numerous processions were observed)). The presence of larvae was checked in every tent collected. Sampled tents were enclosed in bags to keep caterpillars from escaping until further manipulation at INRA two days later. At INRA, individual tents were immediately transferred to pots containing 15 cm of dry sand, capped with mesh netting, and left untouched under a shelter in outdoor conditions until pupation. Fresh branches of black pine were supplied weekly in case some larvae still needed to feed before pupation.

When larvae buried themselves into the sand in all pots, and before first summer emergences, the sand was sorted to collect individuals that survived pupation and transfer them to well multidishes (22 mm diameter). A total of 466 living pupae were obtained, and split into two different conditions: 238 pupae were transferred to a thermal regime consisting of a fluctuation between 10°C and 14°C (8 and 15 hours, respectively, with 30 min gradients), while 228 pupae were transferred to a regime consisting of a fluctuation between 18°C and 22°C (same periodicity). All pupae were kept in the dark.

- **Analysis**

*i. Marcillac-en-Vallon*

About 50% of pupae from this population produced adults on both summers since the time of collection, and few pupae now remain in extended diapause. Table 1 summarizes the number of emerged, diapausing and dead individuals in the laboratory samples. Mortality was first counted in May 2014, before the first emergence peak, but some of the living individuals were transferred to another condition (outdoor) before the development cycle was completed. Consequently, the mortality measured in May 2014 would have been overestimated if compared to laboratory individuals only. Therefore, we had to correct this mortality count by dividing by two the number of dead pupae found in the bags that were eventually split into field and laboratory samples. This corrected initial mortality can thus be compared to the number of individuals that emerged or entered diapause in the laboratory during the first year. In 2016, mortality was specifically monitored on all laboratory individuals and no other individuals were transferred to the field condition, thereby making such correction useless.

**Table 1.** Status of pupae from Marcillac-en-Vallon caught in Écopièges in 2014. Mortality was initially checked before the first emergence peak, and then during winter (February) when no emergences are expected. Emergence counts are the sum of emergences during the summer peak and presumably maladaptive delayed emergences observed later (during the winter mortality check).

| **Winter** | **Emerged (incl. failures)** | **Extended diapause** | **Mortality** |
| --- | --- | --- | --- |
| 2015 | 497 + 9 missing – *50.25 %* | 98 – *9.71 %* | 404 – *40.04 %* |
| 2016 | 48 + 10 missing – *51.79 %* | 28 – *25.00 %* | 26 – *23.21 %* |

*ii. Vienne-en-Val*

Pupae from this population produced adults on the first summer and very few individuals remained in extended diapause when survival was checked in January 2016.

**Table 2.** Status of pupae from Vienne-en-Val caught in Écopièges in 2015.

| **Winter** | **Emerged** | **Extended diapause** | **Mortality** |
| --- | --- | --- | --- |
| 2016 | 737 – *44.21 %* | 25 – *1.49 %* | 905 – *54.29 %* |

**Greece (Markalas 1989)**

Markalas S. 1989. Influence of soil moisture on the mortality, fecundity and diapause of the pine processionary moth (*Thaumetopoea pityocampa* Schiff.). J. Appl. Entomol. 107: 211–215.

- **Temperature data**

Retrieved from the document.

- **Sampling**

One cohort (1984). When the last stage larvae of the pine processionary moth began to leave the trees to pupate in the soil (10-15 April 1984), mature nests were collected. The healthy larvae of each nest were separated into 7 equal groups. Each treatment was consisted of a population with the same genetic traits and developmental conditions. Each treatment was composed of 3 pot-cages with 300-400 larvae placed in each pot for pupation. For the treatment 1, only two cages were used. After the penetration of all the larvae into the soil (25/04/1984), the topsoil of the cages was cleaned of all debris and the number of larvae found dead was counted. The emerged moths were collected every three days and for the female, the number of eggs in their ovaries was counted.

Data emergence and mortality from 1984 to 1988

| Simulated region | N. pupated larvae | N. emerged moths (1984) | % Emergence (1984) | N. emerged moths (1985) | % Emergence (1985) | % Mortality (1984-1988) |
| --- | --- | --- | --- | --- | --- | --- |
| Absolute dryness | 629 | 136 | 21.62 | 113 | 17.97 | 60.41 |
| Athens | 1037 | 198 | 19.09 | 92 | 8.87 | 72.03 |
| Thessaloniki | 1029 | 133 | 12.93 | 29 | 2.82 | 84.26 |
| Ioannina | 1026 | 42 | 4.09 | 3 | 0.29 | 95.61 |
| Much moister | 868 | 35 | 4.03 | 0 | 0 | 95.97 |
| Thessaloniki | 878 | 118 | 13.44 | 23 | 2.62 | 83.94 |
| Thessaloniki | 870 | 74 | 8.51 | 10 | 1.15 | 90.34 |

- **Analysis**

The major mortality factor of the pine processionary moth pupae was the attack by various fungal species. Soil temperature and soil moisture were the factors which influenced the fungal activity in the soil. It seems that the major factor responsible was the soil moisture which was directly related to the amount of simulated rain and may change from place to place and from year to year. The point is to see what is the influence of soil moisture on mortality, fecundity and diapause in pine processionary moth.

When the soil was absolute dryness the rate of mortality was up to 60%, but during the experiment the action of predator wasn’t possible, meaning under real conditions the mortality rate would have been higher. The mortality increased with the soil moisture (up to 96% in the higher soil moisture treatment).

The rate of emergence was higher when the dry period prevailed right after the pupation (May-June) rather than later (July-August). There was a significant decrease of the rate of diapause with the increase of soil moisture. In the moist soil, the rate of diapause was 0%.

During the second year of emergence, 27% more males emerged compared to females. The number of female in prolonged diapause was lower than the number of male. The mortality during the prolonged diapause was higher in female than in male.

The prolonged diapause lasted for only one additional year.

**Israel (Halperin 1969 and 1990)**

Halperin J. 1969. Prolonged pupal diapause in *Thaumetopoea wilkinsoni* Tams. Z. ang. Entomol. 64: 62-64.

Halperin J. 1990. Life history of *Thaumetopoea wilkinsoni* in Israel. J. Appl. Entomol. 110: 1-6.

- **Temperature data**

Retrieved from the documents.

- **Sampling and analysis**

1. Cohorts 1963 and 1964 at Eshtaol

Larvae in the last instar were collected from trees in March and put into wire netting cages (2.1x1.1x0.9 m) in the pine forest. Pine branches were introduced into the cages to provide food to the larvae. After the larvae entered the soil to pupate, the top soil of the cage was cleaned.

There were two cages:

- Cage A: On 18/03/1963, 1500 larvae were collected at Ramat Gan (Coastal plain), 1420 larvae entered the soil during the first half of April for pupation (~80 larvae died before entering the soil).
- Cage B: On 02/03/1964, 800 larvae were collected at Eshtaol, 750 larvae entered the soil during the second half of March for pupation (~50 larvae died before entering the soil).

Observations of the emergence were made at 2-5 day intervals from September to November of each year, and at longer intervals during the following 9 months. The experiment lasted 6 years, from 1963 to 1968 for the cage A and from 1964 to 1968 for the cage B.

| Cage | Years | Date of emergence | N. males | N. females | N. adults | % Emergence |
| --- | --- | --- | --- | --- | --- | --- |
| A | 1963 | 25/09-21/10 | 149 | 418 | 567 | 39.9 |
|  | 1964 | 24/09-03/10 | 0 | 8 | 8 | 0.6 |
|  | 1965 | 20/09-09/10 | 6 | 21 | 27 | 1.9 |
|  | 1966 | 19/09-24/10 | 6 | 8 | 14 | 1 |
|  | 1967 | 26/09-24/10 | 15 | 23 | 38 | 2.7 |
|  | 1968 | 20/09-01/10 | 0 | 6 | 6 | 0.4 |
| B | 1964 | 11/09-08/10 | 34 | 37 | 71 | 9.5 |
|  | 1965 | 14/09 | 0 | 1 | 1 | 0.1 |
|  | 1966 | / | 0 | 0 | 0 | 0 |
|  | 1967 | 14/09-09/10 | 11 | 10 | 21 | 2.8 |
|  | 1968 | 01/09-01/10 | 6 | 24 | 30 | 4 |

Total number of moths at cage A: 660, mortality 760 (53.5%)

Total number of moths at cage B: 123, mortality 627 (83.6%)

Considering the mortality, the rate of prolonged diapause was 6.5% in cage A and 6.9% in cage B.

The rate of emergence was higher in the first year (39.9% in cage A and 9.5% in cage B) and then varied (from 0.4 to 2.7% in cage A and from 0 to 2.8% in cage B). The period of emergence was fairly constant between years and between cages. High mortality rate was observed, especially in cage B.

1. Cohorts 1969 and 1970

Larvae in the last instar were collected from trees or from processions in March and put into wire netting cages (1x1x0.7 m) in the pine forest. Pine branches were introduced into the cages to provide food. Emergences were checked over time.

Two sites:

- Ramallah (850 m): 500 larvae collected in March 1969
- Ramat Gan (50 m): 336 larvae collected in March 1970

| Site | Years | Date of emergence | N. males | N. females | N. adults | % Emergence |
| --- | --- | --- | --- | --- | --- | --- |
| Ramallah | 1969 | 28/08 | 1 | 0 | 1 | 0.2 |
|  | 1970 | 09/09-19/09 | 3 | 1 | 4 | 0.8 |
|  | 1971 | 29/08-12/09 | 4 | 6 | 10 | 2 |
|  | 1972 | 18/08-06/10 | 41 | 14 | 55 | 11 |
|  | 1973 | 06/09-02/10 | 14 | 7 | 19 | 3.8 |
|  | 1974 | 01/09-29/09 | 15 | 27 | 42 | 8.4 |
|  | 1975 | 02/09-27/09 | 53 | 39 | 92 | 18.4 |
|  | 1976 | 03/09-02/10 | 14 | 12 | 26 | 5.2 |
|  | 1977 | 05/09-28/09 | 6 | 10 | 16 | 3.2 |
|  | 1978 | 10/09 | 0 | 1 | 1 | 0.2 |
|  | 1979 | / | 0 | 0 | 0 | 0 |
| Ramat Gan | 1970 | 24/09-09/11 | 92 | 81 | 173 | 51.5 |
|  | 1971 | 27/09-05/10 | 1 | 3 | 4 | 1.2 |
|  | 1972 | 22/09-14/10 | 19 | 13 | 32 | 9.5 |
|  | 1973 | 24/09-14/10 | 6 | 2 | 8 | 2.4 |
|  | 1974 | 20/09-18/10 | 19 | 16 | 35 | 10.4 |
|  | 1975 | 29/09 | 1 | 1 | 2 | 0.6 |
|  | 1976 | 25/09-23/10 | 5 | 2 | 7 | 2.1 |
|  | 1977 | / | 0 | 0 | 0 | 0 |

Total number of moths at Ramallah: 266, mortality: 234 (46.8%)

Total number of moths at Ramat Gan: 261, mortality: 75 (22.3%)

Considering the mortality, the rate of prolonged diapause was 53% at Ramallah and 26.2% at Ramat Gan. The emergence during the first year was very low at Ramallah with a rate of 0.2%. On the contrary at Ramat Gan the emergence the first year was 51.5%.

**Italy Calbarina (Battisti et al. 2000)**

Battisti A., Bernardi M., Ghiraldo C., 2000. Predation by the hoopoe (*Upupa epops*) on pupae of *Thaumetopoea pityocampa* and the likely influence on other natural enemies. BioControl, 45: 311-323.

- **Temperature data**

Retrieved from the documents.

- **Sampling**

Four cohorts (1993 to 1996).

1. Diapausing pupae

Before the pupating processions, at the end of the winter, a few, previously known pupation sites were dug out in search of cocoons. The diapausing pupae were then collected and kept individually in ventilated vials under outdoor conditions and protected from rain. The sampling concerned individuals belonging to more than one diapausing cohort, depending on the duration of the prolonged diapause. The sampling was repeated across three years (1992, 1993, 1994), marked in the table as ‘only pd previous cohorts’.

1. Annual and diapausing pupae
2. After the pupating processions, in late spring, a few, pupation sites were dug out in search of cocoons. The pupae were then collected and kept individually in ventilated vials under outdoor conditions and protected from rain. The sampling concerned individuals belonging to the current cohort and those diapausing from previous cohort. The sampling was limited to the 1993 cohort, marked in the table as ‘annual+pd previous cohorts’.

In the following cohorts (1994, 1995, 1996) the analysis concerned pupation sites that were emptied from pupae in the previous years for sampling. By this way the sampling concerned exclusively the individuals originated with the given cohort, indicated in the table as ‘annual+pd this cohort’.

| Calbarina Padova - Summary of the cohorts | | |  | |  |  |  |
| --- | --- | --- | --- | --- | --- | --- | --- |
|  |  |  | Individuals | | |  |  |
| Cohort | contains | pupae | em y1 | em y2 | | em y3 | dead |
| 1992 | only pd previous cohorts | 263 | - | 97 | | 5 | 161 |
| 1993 | ann+pd previous cohorts | 294 | 32 | 28 | | 2 | 232 |
| 1993 | only pd previous cohorts | 239 | - | 148 | | 3 | 88 |
| 1994 | ann+pd this cohort | 139 | 44 | 5 | | 0 | 90 |
| 1994 | only pd previous cohorts | 142 | - | 4 | | 0 | 138 |
| 1995 | ann+pd this cohort | 462 | 144 | 4 | | 0 | 314 |
| 1996 | ann+pd this cohort | 495 | 81 | 11 | | 0 | 403 |
|  |  |  |  |  | |  |  |
|  |  |  | % |  | |  |  |
| Cohort | contains | pupae | em y1 | em y2 | | em y3 | Dead |
| 1992 | only pd previous cohorts | 263 |  | 36.9 | | 1.9 | 61.2 |
| 1993 | ann+pd previous cohorts | 294 | 10.9 | 9.5 | | 0.7 | 78.9 |
| 1993 | only pd previous cohorts | 239 |  | 61.9 | | 1.3 | 36.8 |
| 1994 | ann+pd this cohort | 139 | 31.7 | 3.6 | | 0.0 | 64.7 |
| 1994 | only pd previous cohorts | 142 |  | 2.8 | | 0.0 | 97.2 |
| 1995 | ann+pd this cohort | 462 | 31.2 | 0.9 | | 0.0 | 68.0 |
| 1996 | ann+pd this cohort | 495 | 16.4 | 2.2 | | 0.0 | 81.4 |

- **Analysis**

There were no pupae extending the diapause to more than 3 years. In addition to the data shown in the table, it was found that moths from prolonged diapause emerged 10-20 days earlier than the annual ones in every cohort, similarly to what has been shown by Salman et al. (2016) for a site of the southern Alps. Sex ratio of annual and diapausing individuals was balanced. The distribution of the pupae in the soil was also explored and significant differences were found among the three selected levels of depth: pupae were more abundant at the depth of 4-8 cm compared to 0-4 and 8-12, with no differences between sexes. Pupae deeper in the soil resulted to be significantly heavier than those in the upper soil layers. Mortality factors were also considered and the major ones were the parasitoids belonging to bombyliids, ichneumonids, and pteromalids, and the predator bird hoopoe (Battisti et al. 2000).

**2. Silandro/Schlanders Bolzano Italy (Salman et al. 2016)**

Salman, M.H.R., Hellrigl, K., Minerbi, S., Battisti, A., 2016. Prolonged pupal diapause drives population dynamics of the pine processionary moth (*Thaumetopoea pityocampa*) in an outbreak expansion area. Forest Ecology and Management 361, 375-381.

- **Temperature data**

Retrieved from the documents.

- **Sampling**

Four cohorts (1999, 2013, 2014, 2015).

**1. Cohort 1999: cage rearing**

Details of the study can be found in Salman et al. (2016). Four cages (2 × 2 × 2 m) in different elevation (822 m, 910 m, 963 m, and 1073 m) were constructed in the south-facing slope of Venosta/Vinschgau valley for the surveillance of the emergence. About 5,000 larvae were added to each cage and pine branches were provided as food. Larvae, upon finishing the feeding, went down the soil for pupation. Leftover branches were removed from the cages and weekly inspections were made for the rest of summer. Emergence were tracked likewise in the following years until 2009 when there was no more emergence for three years and no more pupae available in the soil.

**2. Cohort 2013: cage and laboratory rearing**

As a continuation of the aforementioned study, we restarted two surveillance cages at two elevations (822 m and 910 m) in 2013. We collected more than 6,000 larvae in the 4^th^ and 5^th^ instar and added them into the cages, providing food ad libitum until they pupated into the soil. Emergence has been recorded until now.

In addition to cage surveillance, we collected 500 pupae from the cages and kept them at room temperature in the laboratory of entomology of University of Padova. In early June, we made a visit to the site and burrowed the soil in both cages. Insects were still in prepupal stage. We collected same amount of them uniformly from both cages. Taking into the laboratory, we cleaned and kept them in the glass vials with cotton stopper on them. Thereafter, we kept them at 22-26°C temperature and inspected regularly until all of them emerged or died.

**3. Cohort 2014: laboratory experiment in controlled temperature**

In February of 2014, we deployed modified version of Ecopiège (<https://www.ecopiege-boutique.com/)> collar traps around 33 pine trees in three different elevations. Ecopiège traps were adjusted in such a way so that the bags for collecting larvae through the pipes remain buried under the soil right beside the trees. When the larvae finished their procession and pupated, we collected the bags while leaving the traps on site for the next year experiment.

Following collection, we processed the pupae under the fume hood. First, we sieved the soil from the bags carefully with a metal soil sieve. Soil clung to separated pupae were then cleaned. Sampling a group of individuals, we removed the cocoons and put them individually into the vials. Vials were kept vertically in the holes of polystyrene trays. We then kept them into two controlled temperatures at 10° C and 22°C for the surveillance.

**4. Cohort 2015: laboratory experiment in controlled temperature**

We followed almost the same method described for 2014 for collecting pupae in Venosta. While pots were used instead of bags for collection, frequency of visit for collecting them was weekly during the procession period from the mid-March to the early May. In each visit, we kept half the number of retrieved pots in the field, while took rest of the pots into the laboratory for further analysis. Pots were put in naturally fluctuating outdoor temperature until the larvae developed into pupae. Once they became pupae, we processed them under the fume hood of laboratory by turns and followed the same method for treating them at 10° C and 22° C.

- **Summary results**

**1. Cohort 1999: cage rearing**

A maximum of at least seven years of prolonged diapause in cage surveillance was observed. Retrieval rate of moths decreased and prolonged diapause increased with increasing elevation. Prolonged diapausing individuals emerge in advance of the annual ones.

Emergence and estimated retrieval rate of moths from the rearing cages established in 1999 at four different elevations (from Salman et al. 2016)

| **Year** | **822 m** | | **910 m** | | **963 m** | | **1073 m** | |
| --- | --- | --- | --- | --- | --- | --- | --- | --- |
|  | No.  emerged  moth | %  emergence | No. emerged moth | %  emergence | No. emerged moth | %  emergence | No. emerged moth | %  emergence |
| 1999 | 9 | 0.18 | 2 | 0.04 | 0 | 0 | 0 | 0 |
| 2000 | 402 | 8.04 | 20 | 0.4 | 13 | 0.26 | 0 | 0 |
| 2001 | 100 | 2 | 25 | 0.5 | 87 | 1.74 | 0 | 0 |
| 2002 | 11 | 0.22 | 241 | 4.82 | 20 | 0.4 | 0 | 0 |
| 2003 | 7 | 0.14 | 406 | 8.12 | 70 | 1.4 | 0 | 0 |
| 2004 | 0 | 0 | 100 | 2 | 7 | 0.14 | 0 | 0 |
| 2005 | 0 | 0 | 38 | 0.76 | 1 | 0.02 | 0 | 0 |
| 2006 | 0 | 0 | 9 | 0.18 | 0 | 0 | 0 | 0 |
| 2007 | 0 | 0 | 0 | 0 | 0 | 0 | 0 | 0 |
| 2008 | 0 | 0 | 0 | 0 | 0 | 0 | 0 | 0 |
| **Retrieval %** | 10.58% | | 16.82% | | 3.96% | | 0% | |

**2. Cohort 2013: cage and laboratory rearing**

Both cages produced adults in 2013 (n=55), 2014 (n=11) and 2015 (n=37). Sex ratio of moths, until now, is biased towards female in the cage at 822 m but male in the cage at 910 m. Cages are expected to produce moths in the coming years.

Emergence in the cages till 2015

| **Year** | **822 m** | |  | **910 m** | |
| --- | --- | --- | --- | --- | --- |
|  | Male | Female |  | Male | Female |
| 2013 | 20 | 24 |  | 5 | 5 |
| 2014 | 0 | 5 |  | 1 | 5 |
| 2015 | 7 | 12 |  | 9 | 9 |

A total of 116 pupae (23%) prolonged diapause into the next year, when all of them emerged or died.

Surveillance result of 500 pupae in the laboratory

| **Year** | **Emerged** | **Extended Diapause** | **Mortality** |
| --- | --- | --- | --- |
| 2013 | 130 (26%) | 116 (23%) | 250 (50%) |
| 2014 | 60 (52%) | 0 | 56 (48%) |
| 2015 | 0 | 0 | 0 |

**3. Cohort 2014: laboratory experiment in controlled temperature**

Population density in the field was low. Procession for pupation started at a time usual for the population. Results are summarized in the following table.

Summary surveillance result of cohort 2014

| **Treatment** | **Year n** | | | | **Year n+1** | | | |
| --- | --- | --- | --- | --- | --- | --- | --- | --- |
|  | No. of Pupae | Moth (%) | PD (%) | Dead (%) | No. of Pupae | Moth (%) | PD (%) | Dead (%) |
| 10°C | 311 | 13 | 24 | 63 | 19 | 0 | 47.4 | 52.6 |
| 22°C | 621 | 57 | 19 | 24 | 67 | 1.5 | 89.6 | 9.0 |

**4. Cohort 2015: laboratory experiment in controlled temperature**

Density in the field can be attributed as medium. Procession for pupation started early. Almost no difference in surveillance traits was observed between laboratory and field samples. However, we found significant difference between two temperature treatments in the laboratory where pupae treated with 10°C temperature since the beginning of pupal development showed high percentage of prolonged diapause opposed to those treated with 22°C.

Summary surveillance result of cohort 2015

| **Treatment** | **Year n** | | | |
| --- | --- | --- | --- | --- |
|  | No. of pupae | Moth (%) | PD (%) | Dead (%) |
| 10°C | 279 | 27.2 | 41.6 | 31.2 |
| 22°C | 2158 | 60.0 | 1.3 | 38.7 |

**3. Aspromonte Calabria Italy (Bonsignore 2016)**

Bonsignore C.P., Manti F., Castiglione E. 2015. Interactions between pupae of the pine processionary moth (*Thaumetopoea pityocampa*) and parasitoids in a *Pinus* forest. Bull. Entomol. Res. doi:10.1017/S0007485315000541

- **Temperature data**

Retrieved from the documents.

- **Sampling**

Three cohorts (2010, 2011, 2012).

| Year | Pupae | Emerged 2010 | Emerged 2011 | Emerged 2012 |
| --- | --- | --- | --- | --- |
| 2010 | 3634 | 1354 | 10 | 11 |
| 2011 | 2018 |  | 603 | 2 |
| 2012 | 646 |  |  | 51 |

- **Analysis**

There were no pupae extending the diapause to more than 3 years. Mortality factors were also considered and the major ones were the parasitoids (Bonsignore et al. 2015).

**Morocco (Graf 1988)**

Graf P. 1988. Rapport d’activités de la section de la protection phytosanitaire des forêts campagne 1988. Direction de la Protection des Végétaux des Contrôles Techniques et de la Répression des Fraudes. Ministère de l’agriculture et de la reforme agraire. Salé, Maroc.

- **Temperature data**

Retrieved from the document.

- **Sampling**

Burying cages were set up in the field at the different forest sites in the infested regions by *T. pityocampa* and a part of the larvae was taken to the ”Centre National des Grandes Luttes de la Direction de la Protection des Végétaux des Contrôles Techniques et de la Répression des Fraudes” at Salé, close to Rabat (see map) under outdoor conditions. The larvae were collected from the ground during the last instar and put in cages to bury.

1. Data from cages in the field

| Site | Elevation (m) | Number of larvae put in cage | Rate of emergence in 1988 | % prolonged diapause | % mortality |
| --- | --- | --- | --- | --- | --- |
| Boutroubay (Ifrane) | 1910 | 600 | 40 | 32.5 | 27.5 |
| Ain Nohra (Ifrane) | 1780 | 1000 | 20.3 | 55.5 | 24.2 |
| *Moyen Atlas region* |  | *1600* | *27.8* | *46.3* | *25.8* |
| Ain Rami (Chaouen) | 500 | 800 | 29 | 21 | 50 |
| Bab Taza (Chaouen) | 820 | 400 | 51 | 0.05 | 49 |
| Bouhachem (Tétouan) | 1157 | 388 | 39 | 26 | 35 |
| Issaguen (A.Hoceima) | 1548 | 400 | 20 | 0.05 | 80 |
| Tizi Ifri (A.Hoc.) | 1800 | 1000 | 51 | / | / |
| *North region* |  | *1988* | *33.5* | *13.5* | *58* |
| El Ghomra (Fès) | 500 | 800 | 35 | 19 | 46 |
| J'Zerhoun (Meknès) | 800 | 1200 | 2.5 | 28.8 | 68.7 |
| Oued Beth (Khémisset) | 250 | 800 | 18.8 | 6.5 | 74.7 |
| *Central region* |  | *2800* | *16.4* | *19.6* | *63.9* |
| Jerrada (Oujda) | 1000 | 1370 | 58.2 | 9.8 | 32 |
| Tafoughalt (Oujda) | 800 | 977 | 18 | 30.4 | 51.6 |
| *Oriental region* |  | *2347* | *49.5* | *14.3* | *36.2* |

1. Data from larvae taken to Salé

| Site origin | Elevation (m) | Number of larvae put in cage | Period of emergence | Rate of emergence in 1988 | % prolonged diapause | % mortality |
| --- | --- | --- | --- | --- | --- | --- |
| Tiflet (Khémisset) | 300 | 1560 | 4/07 - 3/10 | 19 | 35.4 | 45.6 |
| Oued Beth (Khémisset) | 250 | 1038 | 3/08 - 8/09 | 3.36 | 8 | 89.7 |
| J’Zerhoun (Meknès) | 500-1000 | 1185 | 18/07 - 7/09 | 8 | 28 | 64 |
| *Central region* |  | *3783* | *4/07 - 3/10* | *12.33* | *26.43* | *61.23* |
| Mgatla | 800 | 180 | 21/07 - 2/09 | 49.4 | / | / |
| El Aioun | 600 | 400 | 4/07 - 23/09 | 49.5 | 10.5 | 40 |
| Ain B.Mathar | 900 | 345 | 11/07 - 25/08 | 20.3 | 8.1 | 71.6 |
| Tafoughalt | 800 | 264 | 11/07 - 19/08 | 16.7 | 16 | 67.3 |
| *Oujda* |  | *1189* | *4/07 - 23/09* | *32.7* | *10* | *57.3* |
| Saka (Taza) | 750 | 820 | 18/07 - 6/09 | 32.2 | 5.9 | 60.9 |
| Tahanaout (Marrak.) | 1000 | 405 | 22/08 - 19/10 | 15.6 | 2.6 | 81.7 |
| Skhour Rham (Settat) | 320 | 210 | 16/08 - 27/09 | 16.7 | / | / |
| Ain Arbi (Ifrane) | 2050 | 431 | ? - 14/06 | 30 | 8.7 | 61.3 |

- **Analysis**

Half of the larvae put in cages died for different causes (no pupation, parasitism, pathogens) from 24.2% in Ain Nohra to 89.7% in Oued Beth. The rate of diapause varied from 0.05% in Bab Taza and Issaguen to 55.5% in Ain Nohra. The correlation between elevation of the larvae’s provenance and the rate of diapause was not significant. The rate of diapause was similar between the larvae on site and those taken to Salé.

The emergence period for adults in Salé lasted a mean of 54 days (36 days for Oued Beth and 91 days for Tiflet). All the results regarding the emergence period from the cages were very heterogeneous and different from the catch period of males by the pheromone traps.

**Spain**

1. ***Granada (Torres-Muros et al. 2017)***

Torres-Muros L, Hódar JA, Zamora R. 2017 Effect of habitat type and soil moisture on pupal stage of a Mediterranean forest pest (*Thaumetopoea pityocampa*). Agric. For. Entomol. DOI: 10.1111/afe.12188

- **Temperature data**

Retrieved from <http://meteo-climat-bzh.dyndns.org> (weather station Granada (37°10'48"N, 03°46'48"W, 567 m).

- **Sampling**

One cohort (2010). The experiment was organized in several squared areas (2x2 m). These areas were prepared in October 2009 and cylinders (30x15 cm) with soil were deployed into the areas. In spring, during the procession period, 9000 larvae were collected and used in two experiments (4500 larvae in each).

|  | Treatment | Type of sampling | N. of larvae |
| --- | --- | --- | --- |
| I.Habitat experiment | Woodland | Larvae from processions | 1500 |
|  | Shrubland | Larvae from processions | 1500 |
|  | Open area | Larvae from processions | 1500 |
| II.Water experiment in open area | Drought | Larvae from processions | 1500 |
|  | Control | Larvae from processions | 1500 |
|  | Watering | Larvae from processions | 1500 |

| Exp. | Treatment | N. of larvae | N. pupae | N. of Emergence (n) | % Emergence n | N. of Emergence (n+1) | % Emergence (n+1) | N. of diapausing pupae (n+2) | % of diapausing pupae (n+2) |
| --- | --- | --- | --- | --- | --- | --- | --- | --- | --- |
| Habitat | Woodland | 1500 | 10.5 | 764 | 8.5 | 7 | 0.1 | 11 | 0.7 |
|  | Shrubland | 1500 | 40.5 |  |  |  |  | * | * |
|  | Open area | 1500 | 2364 |  |  |  |  | 318 | 5.3 |
| Water | Drought | 1500 |  |  |  |  |  |  |  |
|  | Control | 1500 |  |  |  |  |  |  |  |
|  | Watering | 1500 |  |  |  |  |  |  |  |

* Data not available

- **Analysis**

In total, 771 moths emerged, of which 764 emerged in the year of pupation (2010). Most of them were from the open area. In the third summer after pupation (2012), there still were some pupae alive in all treatments (0.7% in woodland to 5.3% in open areas) but the number was low and the experiment ended in 2012.

From the rate of emergence in the year of pupation and the rate of diapausing pupae, it was possible to calculate for all treatments together the following rates:

- 87.8% of mortality,
- 8.5% of emergence,
- 3.7% of prolonged diapause.

Concerning the water experiment, no difference of emergence between the treatments was found. About the habitat experiment, differences were observed, indeed there was no emergence in woodland, a little proportion in shrubland and the most of the emergence were in open area.

1. ***Valencia (Lopez Sebastian, 2014)***

López Sebastián E. 2014. Estudio de la dinámica poblacional del lepidóptero *Thaumetopoea pityocampa* (Denis & Schiffermüller, 1775) en la provincia de Valencia. Universitat de Valencia, Burjasot (Valencia).

- **Temperature data**

Retrieved from <http://meteo-climat-bzh.dyndns.org> (weather station Valencia (39°28'50"N, 00°21'59"W, 11 m).

- **Sampling**

1. Natural pupation

During the procession period (March-April), the naturals pupation sites were recorded and the observations were done in the field.

1. Traps on trees

Traps were put around the trunk in order to catch the larvae when they left the trees. The larvae were in this case forced to pupate in the soil used to fill the traps (see figure). Two months after the procession (in June-July), cocoons were collected.

All the data in the table below are pooled for the two years of sampling (2000 and 2001) and both methods.

| Site | Elevation (m) | N. of pupae | % Emergence | % Diapause | % Mortality |
| --- | --- | --- | --- | --- | --- |
| Los Serranos | 1238 | 88 | 28.1 | 19.7 | 52.2 |
| Sierra Calderona | 300 | 49 | 82.3 | 3.6 | 14.1 |

- **Analysis**

The rate of emergence the current year was quite high at La Sierra Calderona with 82.3% against 28.1% at Los Serranos. The rate of diapause was higher in Los Serranos (19.7%) than in La Sierra Calderona (3.6%). A difference was observed (for both sites) between years, indeed the number of pupae in diapause was higher in 2001 than in 2000.

At la Sierra Calderona, the diapause was extended for 3 years, with 80% of the diapausing pupae (=80% of the 19.7%) emerged after one year of diapause.

At Los Serranos, the diapause was extended for 6 years, with 94% of the diapausing pupae emerged during the first 3 years and 40% the first year of prolonged diapause.

Regarding the mortality of the pupae, 31.2% in Los Serranos and 10.2% in La Sierra Calderona were caused by parasitism. And 21% in Los Serranos and 3.9% in La Sierra Calderona were caused by others factors.
